# The Longevity of the Ancients

**Published:** 1882-03

**Authors:** 


### THE LONGEVITY OF THE ANCIENTS.

CAN man reach and pass the age of 100 years? is a question concerning which physiologists have different opinions. Buffon was the first one in France to raise the question of the extreme limit of human life. In his opinion, man, becoming adult at sixteen, ought to live to six times that age, or to ninety-six years. Having been called upon to account for the phenomenal ages attributed by the Bible to the patriarchs, he risked the following as an explanation: "Before the flood the earth was less solid, less compact than it is now. The law of gravitation had acted for only a little time; the productions of the globe had less consistency, and the body of man, being more supple, was more susceptible of extension. Being able to grow for a longer time, it should, in consequence, live for a longer time than now." The German Heusler has suggested on the same point that the ancients did not divide time as we do. Previous to the age of Abraham, the year, among some people of the East, was only three months, or a season; so that they had a year of spring, one of summer, one of fall and one of winter. The year was extended so as to consist of eight months after Abraham and of twelve months after Joseph. Voltaire rejected the longevity assigned to the patriarchs in the Bible, but accepted without question the stories of the great ages attained by some men in India, where, he says, "It is not rare to see old men of 120 years." The eminent French physiologist, Flourens, fixing the complete development of man at twenty years, teaches that he should live five times as long as it takes him to become an adult. According to this author the moment of a completed development may be recognized by the fact of the junction of the bones with their apophyses. This junction takes place in horses at five years, and the horse does not live beyond twenty-five years; with the ox at four years, and it does not live over twenty years; with the cat at eighteen months, and that animal rarely lives over ten; with the man it is effected at twenty years, and he only exceptionally lives over 100 years. The same physiologist admits, however, that human life may be exceptionally prolonged under certain conditions of comfort, sobriety, freedom from care, regularity of habits and observance of the rules of hygiene, and he terminates his interesting study of the last point ("De la Longevité Humaine") with the aphorism, "Man kills himself rather than dies."

